# Supplementary material for: Effectiveness of a Dental Intervention to Improve Oral Health among Home Care Recipients: A Randomized Controlled Trial
Source: Int J Environ Res Public Health. 2021 Sep 3;18(17):9339. doi: 10.3390/ijerph18179339 (PMC8430536; doi:10.3390/ijerph18179339)
Supplement: Supplementary file 1 [file ijerph-18-09339-s001.zip › ijerph-1317345-supplementary.pdf]

**Supplementary Material**

# Effectiveness of a dental intervention to improve oral health among home care recipients: a randomized controlled trial

**Jonas Czwikla, Alexandra Herzberg, Sonja Kapp, Stephan Kloep, Heinz Rothgang, Ina Nitschke, Cornelius Haffner, Falk Hoffmann**

Correspondence: Dr. Jonas Czwikla
University of Bremen
SOCIUM Research Center on Inequality and Social Policy
Mary-Somerville-Straße 5
28359 Bremen, Germany

Email czwikla@uni-bremen.de

## Part A: Primary Outcome assessed at $t_1$ (results from the subgroup analysis)

### Individuals receiving only informal care

The total Oral Health Assessment Tool (OHAT) mean score in the treatment group (TG) was significantly lower compared to the control group (CG) (2.66 [standard deviation (SD) 2.66] vs. 3.36 [SD 2.42], p=.0108).

The percentage distribution of the total OHAT scores also differed significantly between the groups: 24.4% vs. 9.8% (total score 0), 34.9% vs. 32.4% (total score 1-2), and 40.7% vs. 57.8% (total score 3+) (p=.0118).

With regard to the individual OHAT categories, scores for “gums and tissues” and “oral cleanliness” were lower among TG participants than among CG participants (**Table S1**).

**Table S1.** Oral Health Assessment Tool (OHAT) scores for participants of the treatment group and participants of the control group (individuals receiving informal care only).

| Category | Treatment group (n=86) | | | Control group (n=102) | | | p-value |
|---|---|---|---|---|---|---|---|
| | 0=healthy % | 1=changes % | 2=unhealthy % | 0=healthy % | 1=changes % | 2=unhealthy % | |
| Lips | 89.5 | 8.1 | 2.3 | 88.1 | 11.9 | 0.0 | .2381 |
| Tongue | 80.0 | 16.5 | 3.5 | 73.5 | 23.5 | 2.9 | .4941 |
| Gums and tissues | 62.4 | 28.2 | 9.4 | 43.1 | 41.2 | 15.7 | **.0314** |
| Saliva | 68.6 | 24.4 | 7.0 | 75.5 | 21.6 | 2.9 | .3657 |
| Natural teeth[a] | 50.0 | 38.6 | 11.4 | 45.6 | 40.0 | 14.4 | .7957 |
| Dentures[b] | 64.9 | 28.1 | 7.0 | 58.6 | 31.0 | 10.3 | .7277 |
| Oral cleanliness | 54.7 | 31.4 | 14.0 | 34.3 | 42.2 | 23.5 | **.0173** |
| Dental pain | 91.9 | 8.1 | 0.0 | 87.1 | 9.9 | 3.0 | .3424 |

**Notes:** Boldface indicates significant differences (p<.05).
Missings (n=1 (lips), n=1 (tongue), n=1 (gums and tissues), n=2 (natural teeth), n=2 (dentures), and n=1 (dental pain)) were not considered.
[a] n=72 (treatment group); n=90 (control group).
[b] n=58 (treatment group); n=59 (control group).

In the linear regression, controlled for sex, age group, LTC grade, and time in days between randomization and $t_1$, the total OHAT score was not significantly lower in the TG compared to the CG (-0.69 [95% confidence interval (CI) -1.49 to 0.08]; p=.0781).

**Individuals also receiving formal care**

The total OHAT mean score did not differ significantly between the TG and CG (3.39 [SD 2.32] vs. 3.17 [SD 2.77], p=.5075).

The percentage distribution of the total OHAT scores in the TG and CG also did not differ significantly between the groups: 15.4% vs. 14.3% (total score 0), 19.2% vs. 37.1% (total score 1-2), and 65.4% vs. 48.6% (total score 3+) (p=.3250).

With regard to the individual OHAT categories, do differences were found (**Table S2**).

**Table S2.** Oral Health Assessment Tool (OHAT) scores for participants of the treatment group and participants of the control group (individuals also receiving formal care).

| Category | Treatment group (n=26) | | | Control group (n=35) | | | p-value |
|---|---|---|---|---|---|---|---|
| | 0=healthy % | 1=changes % | 2=unhealthy % | 0=healthy % | 1=changes % | 2=unhealthy % | |
| Lips | 92.3 | 7.7 | 0.0 | 71.4 | 22.9 | 5.7 | .1433 |
| Tongue | 76.0 | 24.0 | 0.0 | 82.9 | 14.3 | 2.9 | .6028 |
| Gums and tissues | 42.3 | 50.0 | 7.7 | 52.9 | 29.4 | 17.6 | .2584 |
| Saliva | 65.4 | 15.4 | 19.2 | 77.1 | 20.0 | 2.9 | .1278 |
| Natural teeth[a] | 45.5 | 45.5 | 9.1 | 59.3 | 18.5 | 22.2 | .1110 |
| Dentures[b] | 45.0 | 40.0 | 15.0 | 66.7 | 16.7 | 16.7 | .2429 |
| Oral cleanliness | 38.5 | 46.2 | 15.4 | 48.6 | 22.9 | 28.6 | .1392 |
| Dental pain | 96.2 | 3.8 | 0.0 | 91.4 | 5.7 | 2.9 | .9999 |

**Notes:** Missings (n=1 (tongue), n=1 (gums and tissues), and n=1 (dentures)) were not considered.
[a] n=22 (treatment group); n=27 (control group).
[b] n=20 (treatment group); n=25 (control group).

In the linear regression, controlled for sex, age group, LTC grade, and time in days between randomization and $t_1$, the total OHAT score was also not significantly lower in the TG compared to the CG (0.56 [95% confidence interval (CI) -0.85 to 1.97]; p=.4259).

## Part B: Secondary Outcomes assessed at $t_1$ (results from the subgroup analysis)

### Individuals receiving only informal care

The total Oral Health Impact Profile (OHIP) mean score did not differ significantly between the TG and CG (9.09 [SD 10.39] vs. 8.35 [SD 11.22], p=.3602).

The percentage distribution of the total OHIP scores in the TG and CG also did not differ significantly between the groups: 30.6% vs. 34.3% (total score 0-1), 21.2% vs. 28.4% (total score 2-5), and 48.2% vs. 37.3% (total score 6+) (p=.2877).

With regard to the individual OHIP items, do differences were found (**Table S3**).

**Table S3.** Oral Health Impact Profile (OHIP) scores for participants of the treatment group and participants of the control group (individuals receiving informal care only).

| Dimension and item | Treatment group (n=85) | | | | | Control group (n=102) | | | | | p-value |
|---|---|---|---|---|---|---|---|---|---|---|---|
| | 0=never | 1=hardly ever | 2=occa-sionally | 3=fairly often | 4=very often | 0=never | 1=hardly ever | 2=occa-sionally | 3=fairly often | 4=very often | |
| | % | % | % | % | % | % | % | % | % | % | |
| **Functional limitation** | | | | | | | | | | | |
| Trouble pronouncing words | 70.6 | 12.9 | 8.2 | 0.0 | 8.2 | 75.5 | 8.8 | 7.8 | 2.9 | 4.9 | .3798 |
| Taste worse | 76.8 | 9.8 | 3.7 | 4.9 | 4.9 | 75.5 | 5.9 | 10.8 | 1.0 | 6.9 | .1571 |
| **Physical pain** | | | | | | | | | | | |
| Painful aching | 71.8 | 10.6 | 8.2 | 7.1 | 2.4 | 63.7 | 10.8 | 13.7 | 3.9 | 7.8 | .2715 |
| Uncomfortable to eat | 57.6 | 9.4 | 16.5 | 10.6 | 5.9 | 64.7 | 9.8 | 5.9 | 7.8 | 11.8 | .1166 |
| **Psychological discomfort** | | | | | | | | | | | |
| Self-conscious | 69.4 | 5.9 | 14.1 | 8.2 | 2.4 | 67.6 | 9.8 | 6.9 | 6.9 | 8.8 | .1536 |
| Tense | 69.0 | 8.3 | 11.9 | 6.0 | 4.8 | 69.6 | 9.8 | 7.8 | 9.8 | 2.9 | .7083 |
| **Physical disability** | | | | | | | | | | | |
| Diet unsatisfactory | 76.2 | 9.5 | 4.8 | 4.8 | 4.8 | 84.3 | 4.9 | 2.0 | 5.9 | 2.9 | .4898 |
| Interrupt meals | 78.8 | 8.2 | 8.2 | 2.4 | 2.4 | 78.2 | 10.9 | 3.0 | 5.0 | 3.0 | .4766 |
| **Psychological disability** | | | | | | | | | | | |
| Difficult to relax | 64.7 | 9.4 | 11.8 | 8.2 | 5.9 | 71.3 | 10.9 | 4.0 | 9.9 | 4.0 | .3287 |
| Been embarrassed | 70.2 | 11.9 | 10.7 | 3.6 | 3.6 | 70.6 | 7.8 | 11.8 | 5.9 | 3.9 | .8603 |
| **Social disability** | | | | | | | | | | | |
| Irritable with others | 76.2 | 8.3 | 10.7 | 4.8 | 0.0 | 81.2 | 8.9 | 5.0 | 3.0 | 2.0 | .4128 |
| Difficulty doing jobs | 67.9 | 11.9 | 4.8 | 6.0 | 9.5 | 82.4 | 4.9 | 2.9 | 5.9 | 3.9 | .1476 |
| **Handicap** | | | | | | | | | | | |
| Life unsatisfying | 63.9 | 7.2 | 7.2 | 16.9 | 4.8 | 66.7 | 7.8 | 10.8 | 7.8 | 6.9 | .3844 |
| Unable to function | 74.1 | 7.1 | 5.9 | 5.9 | 7.1 | 84.3 | 3.9 | 5.9 | 3.9 | 2.0 | .3242 |

**Notes:** Missings (n=3 (taste worse), n=1 (tense), n=1 (diet unsatisfactory), n=1 (interrupt meals), n=1 (difficult to relax), n=1 (been embarrassed), n=2 (irritable with others), n=1 (difficulty doing jobs), and n=2 (life unsatisfying)) were not considered.

In the linear regression, controlled for sex, age group, LTC grade, and time in days between randomization and $t_1$, the total OHIP score was also not significantly lower in the TG compared to the CG (-0.52 [95% confidence interval (CI) -3.78 to 2.73]; p=.7514).

Regarding the periodontal situation, the prevalence of any periodontal problems in the TG was significantly lower than in the CG (77.0% vs. 95.4%, p=.0006). The prevalence of periodontitis did not differ between TG and CG participants (32.4% vs. 45.4%, p=.0955).

In the logistic regressions, controlled for sex, age group, LTC grade, and time in days between randomization and $t_1$, the odds ratio for periodontal problems for TG vs. CG participants was 0.13 (95% CI 0.04 to 0.49; p=.0022), whereas the odds ratio for periodontitis was 0.96 (95% CI 0.46 to 2.03; p=.9194).

## Individuals also receiving formal care

The total Oral Health Impact Profile (OHIP) mean score did not differ significantly between the TG and CG (8.37 [SD 8.13] vs. 6.91 [SD 8.35], p=.3253).

The percentage distribution of the total OHIP scores in the TG and CG also did not differ significantly between the groups: 22.2% vs. 34.3% (total score 0-1), 25.9% vs. 20.0% (total score 2-5), and 51.9% vs. 45.7% (total score 6+) (p=.5713).

With regard to the individual OHIP items, scores for difficulty doing jobs" and "unable to function" were higher among TG participants than among CG participants (**Table S4**).

**Table S4** Oral Health Impact Profile (OHIP) scores for participants of the treatment group and participants of the control group (individuals also receiving formal care).

| Dimension and item | Treatment group (n=27) | | | | | Control group (n=35) | | | | | p-value |
|---|---|---|---|---|---|---|---|---|---|---|---|
| | 0=never | 1=hardly ever | 2=occa-sionally | 3=fairly often | 4=very often | 0=never | 1=hardly ever | 2=occa-sionally | 3=fairly often | 4=very often | |
| | % | % | % | % | % | % | % | % | % | % | |
| **Functional limitation** | | | | | | | | | | | |
| Trouble pronouncing words | 84.6 | 11.5 | 3.8 | 0.0 | 0.0 | 74.3 | 11.4 | 8.6 | 0.0 | 5.7 | .6903 |
| Taste worse | 70.4 | 11.1 | 3.7 | 11.1 | 3.7 | 74.3 | 5.7 | 11.4 | 2.9 | 5.7 | .5439 |
| **Physical pain** | | | | | | | | | | | |
| Painful aching | 63.0 | 11.1 | 11.1 | 11.1 | 3.7 | 54.3 | 5.7 | 20.0 | 14.3 | 5.7 | .8136 |
| Uncomfortable to eat | 51.9 | 14.8 | 14.8 | 11.1 | 7.4 | 71.4 | 0.0 | 11.4 | 5.7 | 11.4 | .1344 |
| **Psychological discomfort** | | | | | | | | | | | |
| Self-conscious | 59.3 | 18.5 | 7.4 | 7.4 | 7.4 | 80.0 | 5.7 | 11.4 | 0.0 | 2.9 | .1416 |
| Tense | 69.2 | 23.1 | 3.8 | 3.8 | 0.0 | 71.4 | 8.6 | 11.4 | 2.9 | 5.7 | .3440 |
| **Physical disability** | | | | | | | | | | | |
| Diet unsatisfactory | 77.8 | 14.8 | 3.7 | 3.7 | 0.0 | 82.9 | 11.4 | 0.0 | 0.0 | 5.7 | .7619 |
| Interrupt meals | 74.1 | 14.8 | 3.7 | 3.7 | 3.7 | 91.4 | 8.6 | 0.0 | 0.0 | 0.0 | .2188 |
| **Psychological disability** | | | | | | | | | | | |
| Difficult to relax | 73.1 | 7.7 | 19.2 | 0.0 | 0.0 | 68.6 | 11.4 | 8.6 | 2.9 | 8.6 | .3927 |
| Been embarrassed | 70.4 | 22.2 | 3.7 | 3.7 | 0.0 | 85.7 | 2.9 | 5.7 | 2.9 | 2.9 | .0794 |
| **Social disability** | | | | | | | | | | | |
| Irritable with others | 81.5 | 11.1 | 3.7 | 3.7 | 0.0 | 94.3 | 0.0 | 5.7 | 0.0 | 0.0 | .1362 |
| Difficulty doing jobs | 61.5 | 7.7 | 15.4 | 11.5 | 3.8 | 88.6 | 2.9 | 2.9 | 0.0 | 5.7 | **.0320** |
| **Handicap** | | | | | | | | | | | |
| Life unsatisfying | 51.9 | 22.2 | 7.4 | 18.5 | 0.0 | 68.6 | 11.4 | 8.6 | 2.9 | 8.6 | .0960 |
| Unable to function | 61.5 | 23.1 | 7.7 | 7.7 | 0.0 | 91.4 | 5.7 | 0.0 | 0.0 | 2.9 | **.0085** |

**Notes:** Boldface indicates significant differences (p<.05).
Missings (n=1 (trouble pronouncing words), n=1 (tense), n=1 (difficult to relax), n=1 (difficulty doing jobs), and n=1 (unable to function)) were not considered.

In the linear regression, controlled for sex, age group, LTC grade, and time in days between randomization and $t_1$, the total OHIP score was also not significantly lower in the TG compared to the CG (0.42 [95% confidence interval (CI) -3.95 to 4.79]; p=.8477).

Regarding the periodontal situation, the prevalence of any periodontal problems (77.3% vs. 80.8%, p=.9999) and the prevalence of periodontitis (45.5% vs. 42.3%, p=.8267) did not differ between TG and CG participants.

In the logistic regressions, controlled for sex, age group, LTC grade, and time in days between randomization and $t_1$, the odds ratio for periodontal problems for TG vs. CG participants could not be estimated because of the low number of individuals without periodontal problems, whereas the odds ratio for periodontitis was 1.36 (95% CI 0.33 to 5.60; p=.6685).

# CONSORT 2010 checklist of information to include when reporting a randomised trial*

| Section/Topic | Item No | Checklist item | Reported on page No |
|---|---|---|---|
| **Title and abstract** | | | |
| | 1a | Identification as a randomised trial in the title | 1 |
| | 1b | Structured summary of trial design, methods, results, and conclusions (for specific guidance see CONSORT for abstracts) | 1 |
| **Introduction** | | | |
| Background and objectives | 2a | Scientific background and explanation of rationale | 1-2 |
| | 2b | Specific objectives or hypotheses | 2 |
| **Methods** | | | |
| Trial design | 3a | Description of trial design (such as parallel, factorial) including allocation ratio | 2 |
| | 3b | Important changes to methods after trial commencement (such as eligibility criteria), with reasons | not applicable |
| Participants | 4a | Eligibility criteria for participants | 2 |
| | 4b | Settings and locations where the data were collected | 2-3 |
| Interventions | 5 | The interventions for each group with sufficient details to allow replication, including how and when they were actually administered | 2-3 |
| Outcomes | 6a | Completely defined pre-specified primary and secondary outcome measures, including how and when they were assessed | 3-4 |
| | 6b | Any changes to trial outcomes after the trial commenced, with reasons | not applicable |
| Sample size | 7a | How sample size was determined | 4 |
| | 7b | When applicable, explanation of any interim analyses and stopping guidelines | not applicable |
| Randomisation: | | | |
| Sequence generation | 8a | Method used to generate the random allocation sequence | 2 |
| | 8b | Type of randomisation; details of any restriction (such as blocking and block size) | 2 |
| Allocation concealment mechanism | 9 | Mechanism used to implement the random allocation sequence (such as sequentially numbered containers), describing any steps taken to conceal the sequence until interventions were assigned | 2 |
| Implementation | 10 | Who generated the random allocation sequence, who enrolled participants, and who assigned participants to interventions | 2 |
| Blinding | 11a | If done, who was blinded after assignment to interventions (for example, participants, care providers, those | 3 |

| | | | |
|---|---|---|---|
| | | assessing outcomes) and how | |
| | 11b | If relevant, description of the similarity of interventions | not applicable |
| Statistical methods | 12a | Statistical methods used to compare groups for primary and secondary outcomes | 4-5 |
| | 12b | Methods for additional analyses, such as subgroup analyses and adjusted analyses | 4-5 |
| **Results** | | | |
| Participant flow (a diagram is strongly recommended) | 13a | For each group, the numbers of participants who were randomly assigned, received intended treatment, and were analysed for the primary outcome | 5-6, Figure 1 |
| | 13b | For each group, losses and exclusions after randomisation, together with reasons | 6, Figure 1 |
| Recruitment | 14a | Dates defining the periods of recruitment and follow-up | 2-3 |
| | 14b | Why the trial ended or was stopped | not applicable |
| Baseline data | 15 | A table showing baseline demographic and clinical characteristics for each group | Table 1 |
| Numbers analysed | 16 | For each group, number of participants (denominator) included in each analysis and whether the analysis was by original assigned groups | 6-8 |
| Outcomes and estimation | 17a | For each primary and secondary outcome, results for each group, and the estimated effect size and its precision (such as 95% confidence interval) | 6-8 |
| | 17b | For binary outcomes, presentation of both absolute and relative effect sizes is recommended | not applicable |
| Ancillary analyses | 18 | Results of any other analyses performed, including subgroup analyses and adjusted analyses, distinguishing pre-specified from exploratory | 7-8 |
| Harms | 19 | All important harms or unintended effects in each group (for specific guidance see CONSORT for harms) | not applicable |
| **Discussion** | | | |
| Limitations | 20 | Trial limitations, addressing sources of potential bias, imprecision, and, if relevant, multiplicity of analyses | 10-11 |
| Generalisability | 21 | Generalisability (external validity, applicability) of the trial findings | 11 |
| Interpretation | 22 | Interpretation consistent with results, balancing benefits and harms, and considering other relevant evidence | 9-10 |
| **Other information** | | | |
| Registration | 23 | Registration number and name of trial registry | 2 |
| Protocol | 24 | Where the full trial protocol can be accessed, if available | not applicable |
| Funding | 25 | Sources of funding and other support (such as supply of drugs), role of funders | 11-12 |

*We strongly recommend reading this statement in conjunction with the CONSORT 2010 Explanation and Elaboration for important clarifications on all the items. If relevant, we also recommend reading CONSORT extensions for cluster randomised trials, non-inferiority and equivalence trials, non-pharmacological treatments, herbal interventions, and pragmatic trials. Additional extensions are forthcoming: for those and for up to date references relevant to this checklist, see www.consort-statement.org.
